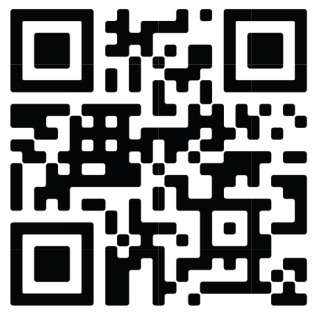# Simplified sperm testing devices: a possible tool to overcome lack of accessibility and inconsistency in male factor infertility diagnosis. An opportunity for low- and middle- income countries

**DOI:** 10.52054/FVVO.13.1.011

**Published:** 2021-03-31

**Authors:** J Onofre*, L Geenen*, A Cox, I Van Der Auwera, F Willendrup, E Andersen, R Campo, N Dhont, W Ombelet

**Affiliations:** Genk Institute for Fertility Technology, Genk, Belgium; Department of Obstetrics, Gynaecology and Infertility, Ziekenhuis Oost Limburg, Genk, Belgium; University of Hasselt, Faculty of Medicine and Life Sciences, Diepenbeek, Belgium; ExSeed Health ApS, Copenhagen, Denmark

**Keywords:** developing countries, low- and middle-income countries, male infertility, smart-phone assisted sperm assessment, semen analysis, variability

## Abstract

**Background:**

Manual semen assessment (MSA) is a key component in a male’s fertility assessment. Clinicians rely on it to make diagnostic and treatment decisions. When performed manually, this routine laboratory test is prone to variability due to human intervention which can lead to misdiagnosis and consequently over- or under- treatment. For standardisation, continuous training, quality control (QC) programs and pricy Computer-Assisted Sperm Analysis (CASA) systems have been proposed, yet, without resolving intra- and inter-laboratory variability. In response, promising simplified sperm testing devices, able to provide cost-effective point-of-care male infertility diagnosis are prospected as a plausible solution to resolve variability and increase access to sperm testing.

**Materials and methods:**

A throughout literature research for semen testing, sperm analysis, smart-phone assisted semen analysis, ‘at-home’ semen testing, male infertility, infertility in developing countries, infertility in low- and middle-income countries (LMIC) and quantitative sperm analysis was performed. A total of 14 articles, specific to ‘at-home’ simplified sperm assessment, were included to treat the core subject.

**Results:**

Continuous training and consistent QC, are sine qua none conditions to achieve accurate and comparable MSA. Compliance does not rule-out variability, nevertheless. Emerging simplified sperm assessment devices are an actual alternative to resolve the lack of standardisation and accessibility to sperm analysis. YO ® , SEEM ® , and ExSeed ® are commercially available, user-friendly smartphone-based devices which can accurately measure volume, sperm concentration (millions/ml) and total motile sperm count. More broadly, by cost-effectiveness, availability, accuracy and convenient application, these devices could effectively select patients for first-line artificial reproduction treatments such as intrauterine insemination.

**Conclusions:**

Accuracy and cost-effectiveness make smart-phone based sperm testing devices a practical and realistic solution to overcome variability in MSA. Importantly, these tools represent an actual opportunity to standardise and improve male subfertility diagnosis and treatment, especially in LMIC. However, before clinical application is possible, guidelines, further testing with special attention on accuracy in washed sperm, availability, cost-benefit and reliability are required.

## Introduction

Male infertility is a global health concern. Directly or indirectly, a male factor contributes for an overall 20 to 70% of cases of infertility with at least 30 million infertile men worldwide (Agarwal et al., 2015). This prevalence varies geographically and is likely to be underestimated, particularly in Africa and Central/Eastern Europe where an overall lack of male infertility assessment facilities, stigma and cultural barriers may lead to underreporting and lack of treatment (Inhorn and Patrizio, 2015; Agarwal et al., 2015; Mehta et al., 2016).

Manual sperm assessment (MSA) remains the ‘gold standard’ to assess male fertility. This routine test considers strict parameters such as semen volume, concentration, motility and morphology, nevertheless, is not sufficient to identify male factor infertility alone (WHO, 2010). It is essential to include a comprehensive medical history (i.e. occupation, influence of environmental and genetic factors, etc...) with an extensive physical examination involving ultrasound imagery of the male genital tract to determine the aetiology, treatment and prognosis of male factor infertility (Kruger et al., 1988; WHO, 2010). This is especially true for low- and middle-income countries (LMIC) where clinicians primarily rely on MSA results to reach a diagnosis and treatment strategy (Franken and Oehninger, 2012).

While sophisticated technologies such as Computer-Assisted Sperm Analysis (CASA) remain underexploited, MSA remains faithful to the application of manual methods, leaving this routine laboratory test, far and wide, susceptible to variability and therefore with an uncertain clinical value. Aiming to reduce variability and to standardize MSA performances, the World Health Organization (WHO) has provided methodological guidelines, reference values and actively recommended the instauration of continuous training and internal and external quality control (QC) programs (Table I) (WHO, 1980, 1987, 1992, 1999, 2010). These efforts have significantly contributed in the establishment of cut-off values for ‘normality’ and an acceptable standardization of methodologies, thus far, not ruling-out intra- and inter-laboratory variability (Franken and Oehninger, 2012; Franken, 2013; Punjabi et al., 2016). Moreover, in a global perspective, the installation of continuous training and QC programs calls into question, due to the outweighed balance between the actual clinical significance of MSA and the arduous logistics and expenses that these programs represent (Keel, 2004; Esteves, 2014; Punjabi et al., 2016).

**Table I:** Minimal threshold values for sperm assessment (WHO, 2010).

Parameter	Value
Total sperm count (million)	> 39
Ejaculate volume (ml)	> 1.5
Sperm concentration (million/ml)	> 14
Total motility (A + B + C + D) (%)	> 40
Progressive motility (%)	> 32
Sperm morphology (%)	> 4

Because of this, plus, with the rapid spread of invasive assisted reproductive technologies (ART), clinicians have lost interest to investigate, diagnose and properly treat the causes of male infertility (Fainberg and Kashanian, 2019). In consequence, inadequate assessment of the causes of male infertility may lead to a situation where the female partner is subjected to invasive, stressful and expensive ART procedures.

Given the general role of MSA as a compelling factor to allocate patients into a treatment strategy, variability must be resolved. For this, emerging user-friendly simplified sperm testing devices are a plausible cost-effective solution to resolve variability and methodological standardization drawbacks (Kanakasabapathy et al., 2017; Kobori, 2019; Oehninger and Ombelet, 2019). More broadly, these devices could represent an ideal alternative to improve access and standardize male infertility diagnosis procedures, namely in LMIC, which in general, lack of funds, equipment and know-how (Franken, 2013; Hammarberg and Kirkman, 2013; Kanakasabapathy et al., 2017).

Here, we provide an outline for MSA and interlink its practical limitations, particularly in LMIC. Finally, we present an overview of existing ‘at- home’ simplified sperm testing devices with special attention to user-friendliness, usability, technique, and collection of data implying the use of these innovative devices in a clinical setting.

## Methods and Materials

**Figure 1:**
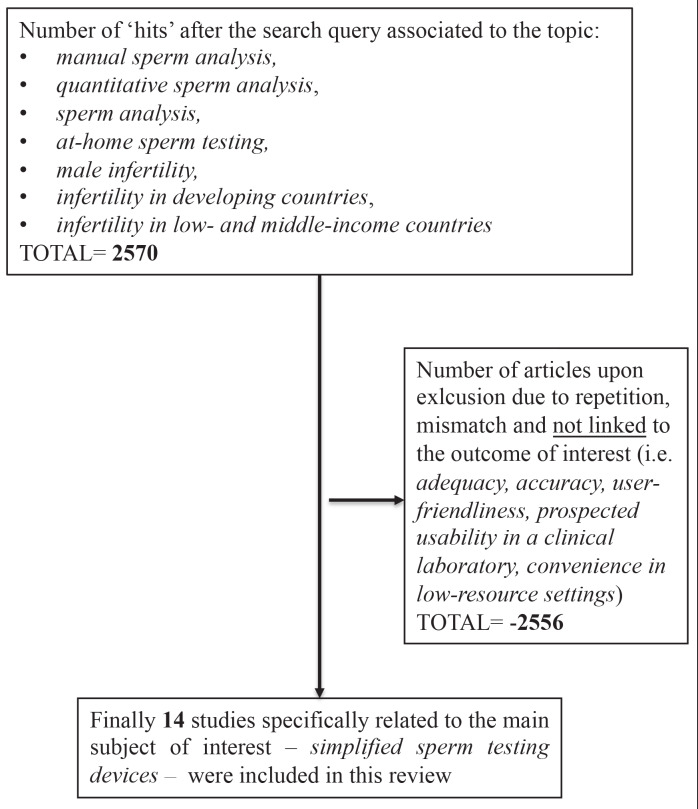
— Analysis diagram for inclusion and exclusion criteria of studies in this review.

A throughout literature research using PubMed, Europe PMC, Web of Science and Google Scholar was conducted. The search terms used were manual sperm analysis, quantitative sperm analysis, sperm analysis, at-home sperm testing, male infertility, infertility in developing countries, infertility in low- and middle-income countries. A first search query led to 2570 papers related to these topics. Repeated hits and articles out of the subject or ones that did not meet our selection criteria (i.e. adequacy, accuracy, user-friendliness, prospected usability in a clinical laboratory, convenience in low-resource settings) were excluded. Finally, we identified 14 articles for a detailed analysis of simplified sperm assessment devices (Figure 1).

## Results

### Current limitations of MSA

Although the assessment of male subfertility has changed dramatically with the introduction of functional sperm testing (e.g. DNA fragmentation index testing; seminal oxidation potential), MSA remains the cornerstone, not only for diagnosis, but also for the choice of treatment in cases of male subfertility (Oehninger and Ombelet, 2019). Therefore, proper application of methodologies and accurate measure of basic sperm parameters (concentration, motility and morphology) when performing MSA is of paramount importance (Ombelet et al., 1997a; Keel, 2004; Douglas et al., 2019).

### Technical difficulties and subjective assessment

With most clinics performing MSA individually, by ‘naked-eye’ and under the subjective judgement of the operator, standardizing the quantification of sperm concentration, motility and morphology remains a quandary (Mortimer e al., 1986; Keel, 2004). Although the WHO (2010) recommends the use of an improved capacity Neubauer haemocytometer to count immobilized (diluted in deionized water) sperm to determine concentration, laboratories overall persist using a Makler counting chamber because of unfamiliarity, habit or practical ease, disregarding the improved accuracy of the recommended Neubauer chamber. Indeed, overgoing volume errors when pipetting (3 - 10μl admitted volume) allowing highly motile sperm to ‘reservoir’ under the counting grid and the risk of assessing artificially increased concentration and motility if a sample’s assessment is performed before its consistent distribution under the chamber’s empty space (inverse correlation between time elapsed at sample inclusion and assessment) are main sources of error, inaccuracy and inter-operator variablity when using a Makler chamber (Walls et al., 2012; Franken, 2013; Björndahl et al., 2016; Tomlinson, 2016; Ahadi et al., 2019; Zuvela and Matson, 2020). In spite of this, the capacity of simultaneously estimate sperm concentration and motility, actual outline of simplified sperm assessment, permitting the successive run-up of several samples in a shorter time, is a practical perk still favouring the use of a Makler chamber in most laboratories, nevertheless.

The evaluation of motility, in spite of its correlation with fertilization and subsequent pregnancy (Ombelet et al, 1997c), remains an actual challenge due to the need for individually distinct between rapid (A) and slow (B) progressive motility (Cooper and Yeung, 2006).

Subjectivity when assessing motility leads to a large margin of overestimation because of the attraction of the eye to movement, especially in specimens with high sperm concentration (Brazil, 2010; Tomlinson et al., 2010). In the fifth edition (2010) of the WHO manual a consistent solution, by signifying the importance of progressive motility (A+B) over the subjective assessment of individual rapid progressive motility (A or B), has been proposed resulting in much lower intra- and inter-laboratory variability (WHO, 2010; Filimberti et al., 2012; Punjabi et al., 2016).

Despite being recognized as the parameter that mostly correlates with the in vivo and in vitro fertilizing ability of sperm (Kruger et al., 1988; Ombelet et al., 1997b; Sallam et al., 2003; Buck et al., 2014;), sperm morphology remains the most subjective parameter to evaluate (Franken, 2013; Oehninger and Ombelet, 2019). The technical difficulties associated to the preparation of samples (i.e. smear and (modified) Papanicolaou staining preparations), but mostly, the difficult interpretation of the strict criteria to draw normal spermatozoa from ‘borderline abnormal spermatozoa’ at scoring are blameworthy of large intra- and inter-observer variability (Ombelet et al., 1998; Brazil, 2010; WHO, 2010; Franken, 2013; Oehninger and Ombelet, 2019). Still, a trend towards reduced variability in morphology assessment was reported, when several laboratories in Belgium continuously adopted the WHO (2010) strict criteria and recommended preparation techniques through continuous training and QC, at a national level (Punjabi et al., 2016).

### Striving QC and training programs

To achieve reliable and comparable results upon MSA, Mortimer et al. (1986) introduced the concept of complementary internal and external QC. The WHO has endorsed and recommended the use of continuous internal and external QC onwards the fourth edition of their manual (WHO, 1999) and has further emphasized its use in their latest edition (WHO, 2010). Although these manuals are recognized as the benchmark for sperm assessment worldwide, several reports highlight failure and inadequacies when implementing recommendations and reference (updated) cut- off values, disclosing an overall poor level of understanding and an overlook on male infertility diagnosis (Ombelet et al., 1997b; Keel, 2004; Álvarez et al., 2005; Cooper et al., 2007; Mallidis et al., 2012; Filimberti et al., 2012; Punjabi et al., 2016; Ahadi et al., 2019). As a consequence, men with suboptimal semen quality are not properly diagnosed and treated, often leading to a common situation where the female partner is subjected to invasive, stressful and expensive ART procedures without consideration for alternative treatment solutions.

In countries applying reimbursement policies (i.e. Belgium, the Netherlands, France), and despite the costs and strenuous logistics behind QC programs, the risk of economic losses that unnecessary or erroneous treatments, in consequence of the biased application of MSA has facilitated the instauration of continuous training and QC programs achieving an overall acceptable harmonization of methodologies, although not entirely ruling-out intra- and inter-laboratory variability (Auger et al., 2000; Punjabi et al., 2016). On the contrary, in countries with partial or inexistent infertility reimbursement policies or without a long-standing experience in in vitro fertilisation (IVF) programs, the instauration of QC is a substantial challenge. Higher drop-out rates, methodological differences and intra- and inter- laboratory variability have been reported (Álvarez et al., 2005; Cooper et al., 2007; Mallidis et al., 2012; Filimberti et al., 2012; Franken, 2013; Mehta et al., 2016; Ahadi et al., 2019).

The instauration of extensive and expensive training and QC programs in laboratories in LMIC appears elusive, nevertheless, as MSA remains the key to male fertility investigation in these settings, continuous training and QC are essential (Deonandan et al., 2012). Franken (2013) in cooperation with the WHO’s Human Reproductive Programme revealed the feasibility and vital role of training and QC programmes in LMIC. Between 1997 and 2013, 16 African and Indian andrology laboratories achieved continuous improvement, development and maintenance of appropriate hands-on skills and results thanks to this initiative, yet not resolving variability when assessing motility and morphology (Franken and Oehninger, 2012; Franken, 2013).

Certainly, the subjective assessment of a videotape (or DVD) and performing sperm assessment at a different temperature than 37°C at the moment of QC monitoring are factors affecting these observations (Álvarez et al., 2005; Filimberti et al., 2012; Daoud et al., 2016).

### Underexploited technical advances

In the evidence that complying to training and QC programs does not entirely exclude variability and error, modern CASA systems, automatically gathering concentration, motion patterns and morphology of sperm, have been put to test (Mortimer et al., 1995). With a bulky design and using a systematic microscope-based image analysis approach, these systems have shown more consistency than MSA, namely on washed human sperm samples, which, unlike neat sperm, typically present high motility and minimal contamination with other cells and debris (Dearing et al., 2014; Mortimer et al., 2015). Despite its usefulness and advantages, parameter settings, used algorithms, as well as professional training, routine calibration and QC exercises are also critical to minimize errors, which if disregarded may lead to significant variation, have limited its clinical application (Holt et al., 1994; Hu et al., 2013; Mortimer et al., 2015; Talarczyk-Desole et al., 2017). Together these drawbacks, plus importantly, its price and running costs, unwarrant its use in small clinics and hospitals in advantage of MSA.

Although sperm concentration, motility, and morphology are the basic components for determining a specimen quality, techniques to analyse the functional characteristics of sperm (e.g. acrosome reaction, capacitation and the integrity of sperm DNA) relying on sophisticated flow cytometry, halo tests or single-cell gel electrophoresis, are under the spotlight. Despite providing valuable information for diagnosis, prognosis and treatment, these functional tests are still considered challenging due to the required technical skills, time and, importantly, expense for the clinic and thus for the patient (Oehninger and Ombelet, 2019). Also, an important lack of consensus over the significance of its results subsists (Practice Committee of the American Society for Reproductive Medicine, 2015).

### Cultural and economic constraints

In many cultures and for many men male infertility is linked to feelings of uneasiness, prejudice (feelings of being less masculine, weak and ineffective) and social stigmatization. These unwarranted conceptions can lead to a situation where, despite being aware of their infertility, men ‘avoid’ to be diagnosed and treated, with exceptional cases where women are individually blamed for the couple’s childlessness resulting in significant social consequences (Folkvord and Odegaard, 2005; Cui, 2010; Dhont, 2011; Wischmann and Thorn, 2013; Inhorn and Patrizio, 2015). With the globalization of ART and health information, men are breaching these limiting barriers with a growing number willing to cooperate in diagnosis and treatment of infertility (Dyer et al., 2004; Dhont, 2013).

Unfortunately, the accountability of inadequate diagnosis and treatment of male infertility is accentuated by economic limitations. With overall andrology testing facilities readily not available, charged prices or due to an impeding lack of funds, equipment, experience and know- how, male infertility diagnosis and testing remains limited, translating to limited access to fertility care (van Balen and Gerrits, 2001; Franken and Oehninger, 2012; Hammarberg and Kirkman, 2013; Bahamondes and Makuch, 2014; Mehta et al., 2016; Ombelet and Onofre, 2019). As a result, many infertile couples suffer from involuntary childlessness with psychological and socio-economic consequences (Barden-O’Fallon, 2005a,b; Inhorn and Patrizio, 2015).

**Figure 2:**
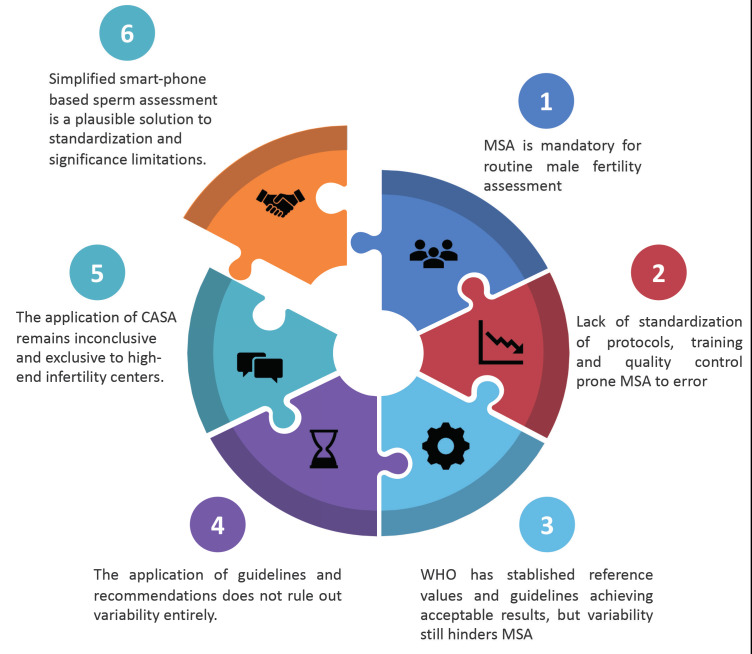
— The introduction of simplified smart-phone based devices to assess sperm in the clinic is a plausible solution to close the loop of lack of standardisation, variability, significance and availability limitations hindering sperm assessment. MSA: Manual Sperm Assessment.

Countering subjectivity, technical inadequacies and access limitations, but also allowing more privacy to the user, newly-developed simplified sperm testing devices may be a cost-effective option to circumvent the aforementioned drawbacks of MSA. Provided accuracy and availability, these devices are ideal candidates to improve access and fluency to male infertility diagnosis, certainly in LMIC. Interestingly, some of these devices have already proven to be reliable for clinical testing of sperm concentration and motility during remote ‘point-of-care’ male infertility screening (Kanakasabapathy et al., 2017). The exploration of their clinical reliability and robustness of results is a worthwhile pursuit to close the loop of lack of standardization, accuracy, and significance of sperm assessment results worldwide (Figure 2).

### Simplified devices for male fertility analysis

Several newly-developed simplified and less costly sperm testing devices are currently available on the market (Kobori, 2019). Interestingly, some of these devices have proven to be reliable in the hands of professionals and non-professionals encouraging their application, not only for ‘at-home’ screening, but also as a point-of-care tool for remote application (Kanakasabapathy et al., 2017)

**Figure 3:**
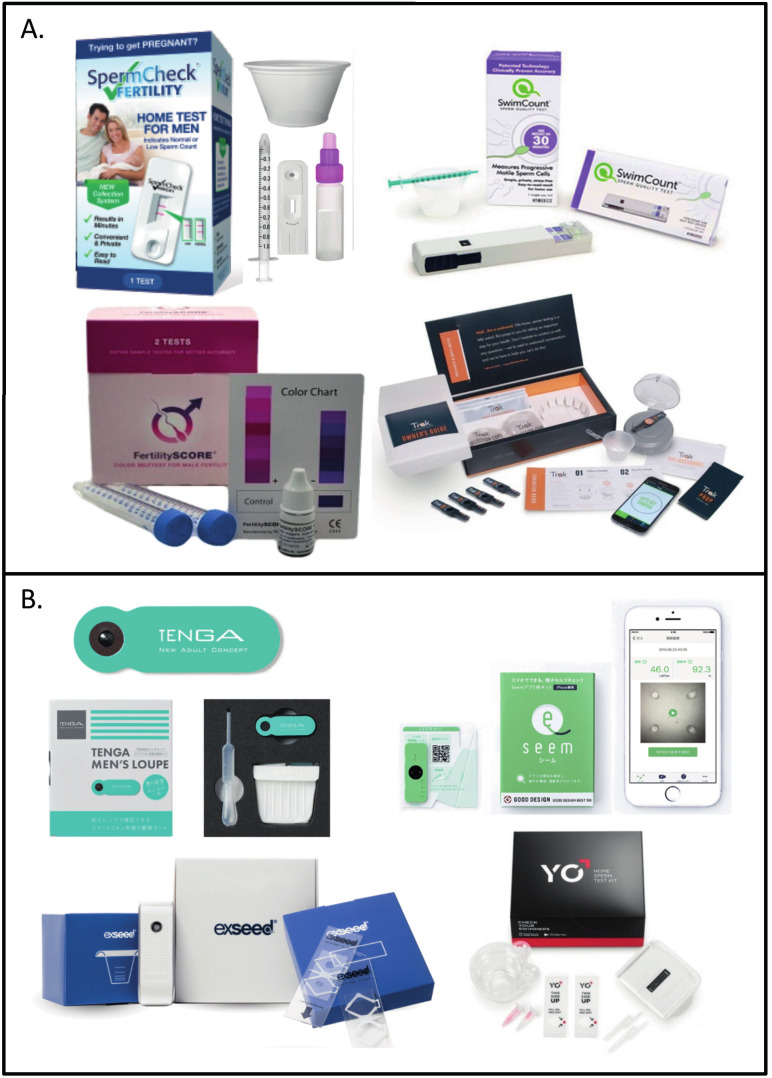
— Simplification, downsizing and cost-effectivenes has been achieved by the implementation of innovative methods including colourimetrie, chromatpgraphic and microfluidics with short-scale centrifugation (A) and smartphone-based microscopy with software-assisted analysis (B). These devises are commercially available, clinically validated for accurate sperm assessment and, importantly, approved by the FDA (SpermCheck ® , Trak ® , SwimCount ® and YO ® ) or CE marked in the EU (YO ® , SwimCount ® , SpermCheck ® and ExSeed ® ).

**Figure 4:**
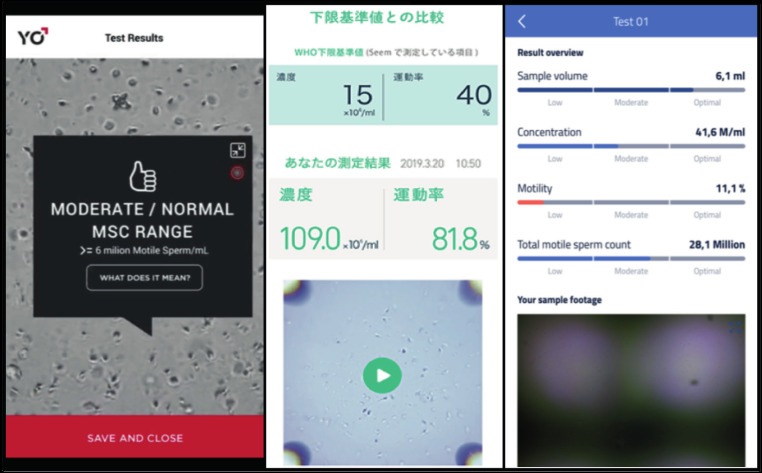
— Smart-phone based sperm assessment is a good alternative for quantitative sperm diagnosis in settings limited by lack of equipment and/or trained personnel. YO ® Sperm Test (A), SEEM ® (B) and ExSeed ® (C) are user-friendly, accurate and cost- efficient candidates to be used in the clinic. They operate using an adaptable magnifitying lense to record a video of the sample to analyze it using a quatification software. Upon testing, SEEM ® and ExSeed ® display an exact quantitative measure (B, C), appropriate for clinical use, whilst the YO ® Sperm Test (A) displays only a qualitative result.

Simplification, downsizing and cost-effectiveness of sperm assessment require the use of innovative methodologies relying on conventional microscopy, chromatographic immunoassays, colourimetric reactions, microfluidics coupled with colourimetric reactions, centrifugation and smartphone-based microscopy (Klotz et al., 2008; Garcia-Laez et al., 2017; Kanakasabapathy et al., 2017; Agarwal et al., 2018; Bar-Chama et al., 2019; Cheon et al., 2019; Yoon et al., 2020). To meet the requirements that, in our experience, would allow the use of these devices in different point-of-care clinical settings, the device should be inexpensive, rapid, easy-to- use and, importantly, able to measure total sperm concentration (millions/ml) and percentage of motile sperm in neat and washed sperm samples following the minimal WHO reference values (WHO, 2010). An overview of different candidate devices reviewed is given in Figure 3, Figure 4, and Table II.

**Table II:** Current and promising options for at-home sperm testing and point-of-care male infertility screening.

Device	Technique	Gathered results	Accuracy (%)	No. of tests per kit	Price per test (usd)^	Point-of-care application	Reference
Micra sperm Test	Microscopy	Concentration, Motility	User dependent	2	22	No advantage over standard MSA	N/A
SpermCheck® Fertility	Chromatographic immunoasay with colourimetric reaction	Concentration 20x10^7^	98	2	20	No, qualitative measure only and expensive	Coppola et al., 2010
SpermCheck® Vasectomy		Concentration > 250,000	Sensitivity: 93 Specificity: 97	2	30		Klotz et al., 2008
SwimCount®		Concentration 5x10^6^- 20x10^6^	95	1	57		Yoon et al., 2020
FertilityScore®		TMSC < 20x10^6^	93	2	12,5		N/A
Track® Male Fertility Testing System	Centrifuge with colourimetric reaction	Concentration (low, moderate, optimal)	95,5 in high concentration samples	2	50		Schaff et al., 2017
Kanakasabapathy device	Smart-phone based	Volume, Concentration, Motility	97,1 Sensitivity: 100 Specificity: 69	N/A	N/A	Yes, but not commercially available	Kanakasabapathy et al., 2017
Tenga® Mens Loupe	Smart-phone based	Volume, Concentration, Motility	Sensitivity: 87,5 Specificity: 90,9	4	3,75	No advantage over standard MSA	Kobori et al., 2016
YO® Home Sperm Test		Volume, TMSC > 6x10^6^	iPhone 7: 98.3 Samsung Gal.S2: 97.2%	2	25	Yes, FDA and CE approved but only qualitative measure available	Agarwal et al., 2018
SEEM®		Volume, Concentration, Motility	96,7	2	22	Yes, FDA and CE approved but limited to certain smartphones only	Cheon et al., 2019
ExSeed® Home Sperm Test		Volume, Concentration, TMSC	96	5	42	Yes, CE approved with convinient use and price	N/A

^ approximate price per test as from online sources, N/A: not available MSA: manual sperm assessment.

### Conventional microscopy

Using a conventional microscope to assess the number and motility of sperm cells at home, such as with the Micra Sperm test, undermining the difficulty of routine sperm analysis, has been proposed. In this process, semen samples can be analysed using graduated chamber microscope slides. For accurate sperm assessment, if performed by non-experienced/non- trained personnel, this technique is highly susceptible to variation because of all the manipulations, plus, in terms of equipment, it replicates to a extend the standard laboratory procedure, hence, not representing an actual simplification.

### Chromatographic immunoassays

Innovative immunoassays permitting a colourimetric reaction upon the binding of sperm-specific monoclonal antibodies are used in assays such as the SpermCheck ® and SwimCount ® . These systems produce a signal in the presence of sperm, only observable above a certain threshold, which can be categorized in a graduated scale displaying an estimation of sperm concentration alone (Figure 3).

SpermCheck® relays on a two-phase solid-phase chromatographic immunoassay within a cassette showing a positive result within a concentration threshold of 2x10^7^ sperm/ml. Men can use this ‘at-home’ test to screen their sperm for normozoospermia or oligozoospermia with a reported accuracy of 98% (Table II) (Coppola et al., 2010). Interestingly, this test is also available for a threshold <250,000 sperm cells (SpermCheckVasectomy ® ) to confirm the efficiency upon vasectomy interventions (Klotz et al., 2008).

The SwimCount ® device presents the advantage of investigating the individuals fertility potential by testing the total motile sperm count (TMSC). The application of sperm ‘swim-up’ in a microfluidic chamber allows to discriminate normal motile spermatozoa with low DNA fragmentation and to displaying these results within a purple scale signal for samples between < 5x10^6^ (light) and > 20x10^6^ (dark) sperm/ml. In terms of accuracy compared to manual counting, the SwimCount ® , which divide results into three categories of estimated concentration, has been reported reliable finding an accuracy of 95% in determining a TMSC <5x10^6^ cells/mL (Figure 3) (Yoon et al., 2020). Similarly, the FertilitySCORE ® measures TMSC based on the sample’s sperm metabolic activity. The signal ranges from blue to pink within a threshold of 20x10^6^ sperm/ml. Comparatively to CASA, this test reports a 93% accuracy (Table II) (Zalata et al.,1995).

More sophisticated microfluidic devices combine a colourimetric reaction with short-scale centrifugation and resistive pulse. The Trak ® device permits the analysis of a defined volume of semen treated via centrifugation setting a visible level gauge, proportional to the concentration of sperm. This concentration can be categorized to three levels (low, normal, high), therefore, limiting its outcome measure by not displaying an exact quantitative result (Table II). The Trak ® system was also tested next to CASA and presented consistent results (Figure 3) (Schaff et al., 2017).

Other microfluidic techniques used to analyse sperm include electrical impedance, oriented sperm swimming, random swimming orientation/ sedimentation, electrical impedance and colourimetric signals. For most, these devices call for extra equipment which increases their cost and makes them less compact (Kobori, 2019; Yu et al., 2018).

### Smart-phone based semen analysis

Downsizing CASA software and hardware to a smartphone’s connectivity and camera is currently the most promising tool to achieve precise sperm counting (Kobori et al., 2016; Sigman, 2016; Agarwal et al., 2018; Kanakasabapathy et al., 2017; Kobori, 2019). Many smart-phone based devices are currently commercially available and clinically validated (Schaff et al., 2017; Agarwal et al., 2018; Bar-Chama et al., 2019).

Originally, to enhance the capacity of the smartphone camera, a ball lens microscope adaptable to a smartphone’s camera could be used as in the Tenga ® Men's Loupe (Figure 3). In this device the ball lens provides a 555X magnification to record a video with the smartphone camera. The user must perform manual assessment risking potential variability and errors due to unexperienced counting. Kobori et al. (2016) reported a strong correlation (87.5% sensitivity and 90.9% specificity) between CASA and Tenga® Men's Loupe, nevertheless.

In a similar fashion, SEEM ® uses a mounted magnifying lens to acquire a video of the sperm and automatically analyses sperm motility and concentration interlinking results to the minimum reference WHO values without the intervention of the user (Figure 4) (WHO, 2010) (Video 1). This device has been compared to CASA and was found to be accurate for both concentration and motility (Table II) (Cheon et al., 2019).

Kanakasabapathy et al. (2017) described the development of a smart-phone based diagnostic assay, integrating microfluidics and image optical sensing enhanced by smartphone capabilities, with the aim to perform remote semen quality testing in high-end and resource-challenged settings. Upon analysis of unwashed, unprocessed liquefied semen samples this device achieved 98% accurate semen quality evaluation based on the WHO guidelines with <5-seconds mean processing time, paving the way of routine point-of-care, low-cost and reliable semen analysis (Table II). Unfortunately, to our knowledge, this system is not currently available in the market.

The YO ® Home Sperm Test permits to connect different models of smartphones to an analysis station where a slide including the sample is inserted. Upon testing, quantitative results are gathered and automatically indicate if the measured concentration of motile sperm is above a certain threshold (Figure 3) (Video 2). This assay uses the smart-phone’s camera an light to assess concentration and motility in the sample to a “low” or “moderate” indication based on the established bottom-end 6x10^6^ sperm/ ml cut-off value by the WHO (WHO, 2010). The YO ® Home Sperm Test has been extensively tested and compared to MSA and to SQA-vision (CASA) for identification of abnormal motile sperm concentration values and accuracy reaching 98.3% and 97.2% when using an Apple iPhone 7 or and Samsung Galaxy S2, respectively. Interestingly, when the device was used by untrained users and trained technicians, both groups had high accuracy and similar results (Agarwal et al., 2018; Bar-Chama et al., 2019).

Consenting a wider range of smartphone models, the ExSeed ® Home Sperm Test uses a docking station to align the camera and enhancing lens, permitting to gather and analyse results through the connectivity of the smartphone by the provided software. Importantly, volume, motility and concentration are accounted for the assessment of sperm using a double chamber slide, therefore offering enhanced precision and replicability (Figure 3, Figure 4) (Video 3). The ExSeed ® device reports a precise absolute quantitative result of basic semen parameters as well as the TMSC, representing a considerable advantage in the perspective of using these devices for first-line male fertility diagnostics in high-end and resource-challenged laboratories.

Importantly, most systems have been approved by the FDA (SpermCheck ® , Trak ® , SwimCount ® and YO ® ) or as In Vitro Diagnostics device in the EU (YO ® , SwimCount ® , SpermCheck ® and ExSeed ® ) in the last few years. Accounting on accuracy, usability and user-friendliness these devices may represent a plausible solution not only for ‘at-home’ testing but also for cost-efficient point-of-care quantitative sperm diagnostics.

## Discussion

Systemic and human errors including technical difficulties, lack of compliance with recommended protocols and guidelines, plus, importantly, the subjective nature of sperm assessment can influence MSA results. Relaying on arduous logistics and high-cost, QC and training remain moreover limited by a the extensive and impassable learning curves. Several reports demonstrate standardization and acceptable results only upon a decade or more from implementation of training and QC programs, being a major pitfall to establish the clinical significance and comparability of MSA results (Punjabi et al., 2015; Álvarez et al., 2005).

When performed by personnel out-of-touch, or out of QC monitoring programs, MSA remains an unreliable indicator of a man's fertility status. This can lead to misdiagnosis and over- or under- treatment with different physical, emotional and financial costs (Baker et al., 1981; Ombelet et al., 1997b; Keel, 2004; Ombelet et al., 2008a; Esteves, 2014; Punjabi et al., 2016). Evidence shows that current recommendations for MSA application still fails to entirely eliminate intra- and inter-laboratory variability from subjectivity and human error (Álvarez et al., 2005; Cooper et al., 2007; Mallidis et al., 2012; Filimberti et al., 2012; Franken, 2013; Mehta et al., 2016; Tomlinson, 2016; Ahadi et al., 2019). Therefore, misdiagnosis related to the erroneous MSA remains an actual risk (Keel, 2004; Franken and Oehninger, 2012; Esteves, 2014).

These errors might not be critical or even resented by patients in countries enjoying reimbursement or sufficient purchasing power to face the costs of infertility diagnosis, but may be deleterious for people, especially in LMIC, who cannot cope with high out-of-pocket medical care costs (Dyer and Patel, 2012).

To ease learning and application, turning to technological advances such as CASA, appears evident. Yet, to this date these systems remain underused, mostly due to its price (Mortimer et al., 1995, 2015; Talarczyk-Desole et al., 2017). Adapting to a growing market, smaller and practical CASA systems, banking their accuracy and consistency on evolutive artificial intelligence (AI) (e.g. Lenshooke X1-Pro Semen Analyser) and capable of immediate morphology examination, are currently gaining market. With listing prices > 12,000$, these remain an expensive diagnostic tool excluding potential users, certainly in LMIC.

Modern accurate, available and cost-effective smart-phone based sperm testing devices, permitting to depict prognostic values such as volume, total sperm concentration and TMSC, in a short time and at a lower cost can overcome variability and access limitations in MSA. With a main frame of development allowing to test users' sperm at the privacy of their home, these devices have the potential to breach uneasiness and disagreement barriers towards male infertility diagnostic. Intuitively, these systems may convey these men to visit a clinic for formal testing, nevertheless, with risk of over- and under- medicalisation by inciting the patient to visit a fertility specialist or by false-negative results when prevailing functional complications exist (Kobori, 2019; Bar-Chama et al., 2019).

On the other hand, applied as a first-line point- of-care male infertility screening tool, these devices have the advantage of requiring little to no training nor maintenance, therefore diminishing the extent of hands-on training and learning curve before accurate application, and report to be highly accurate within ranges, between 16,000 to 1,644 million cells, as generally presented in manual counting data analysis (own data not published), but also when compared to MSA and CASA systems and when used by untrained users and trained technicians (Agarwal et al., 2018; Bar- Chama et al., 2019; Kobori, 2019).

Despite fulfilling usability, accuracy and cost criteria, by its convenience and low-manufacturing costs (< $5 material cost), the device produced by Kanakasabapathy et al. (2017), specifically developed for point-of-care application, appears not to be commercially available. Accounting on usability, user-friendliness and accuracy, other smartphone-based devices like SEEM ® , YO ® and ExSeed ® , are also promising candidates for point- of-care clinical applications. In terms of usability, YO ® and ExSeed ® present the advantage of accepting several smartphone models. Importantly, by its quantitative display the ExSeed ® presents a direct quantitative measure upon testing (Figure 4). Also, it offers more testing slides in one kit, each slide using two counting chambers with the possibility of testing in duplicate, suggesting an enhanced accuracy. Yet, to endorse the expected results with the ExSeed ® device, a clinical validation study comparing it to manual or automated sperm count is lacking. Alternatively, the YO ® Home Sperm Test currently offers only a qualitative result (normal, average, low) in its commercially available version, which is unpractical for this application. However, it has the possibility to display a quantitative measure as shown elsewhere (Figure 4) (Agarwal et al., 2018; Bar-Chama et al., 2019).

With prices per test ranging from 42 $ for ExSeed over to 22 - 25 $ for SEEM and YO,these devices undoubtedly represent a cost benefit alternative reducing short-to medium term expenses. Comparatively, at the Ziekenhuis Oost Limburg, treating about 636 samples/year in 2019 for diagnostics alone, prices for reagents and disposables reached 3.9€ /sample and total prices for equipment of 14688 €, leading to a total annual expense of ~17168 €/year excluding taxes and technician hourly salary expenses (~1600hours/ year) (own data not published), for a sameprice and with a reduced processing time, a total of 408 tests using the ExSeed or 780 using the SEEM could be performed. Additionally, both YO ® Home Sperm Test and ExSeed ® offer the possibility to reuse their docking device, which is the considerable cost. They also provide new consumables for continued testing, therefore providing additional cost-effectiveness.

In spite of these advantages, a common denominator between all these devices remains the limited outcomes measured: apart from volume, concentration, and motility nothing else is assessed. As mentioned before, these parameters alone are not sufficient to disclose male infertility and morphology scoring is not available although its value might be important (Kruger et al., 1988; Ombelet et al., 1997b; Sallam et al., 2003; Kanakasabapathy et al., 2017; Douglas et al., 2019). Additionally, as for CASA, technical difficulties such as size-based cell misidentification, when measuring sperm concentration in samples presenting higher-than- average numbers of non-sperm cells (i.e. white blood cells) and debris of similar size to the sperm head, have also been reported. Moreover, for all devices, verification for use with washed sperm is still required (Kanakasabapathy et al., 2017; Kobori et al., 2016; Samuel et al., 2018). Also, to our knowledge, there is no data on the influence of these systems in the attitudes and views of men in LMIC to test their fertility, nor on their usability in point-of-care routine.

**Figure 5:**
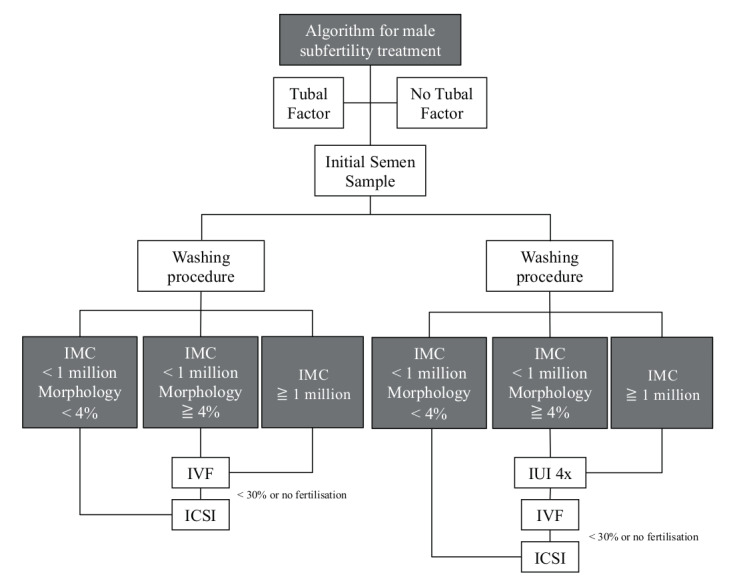
— Fertility strategy

Despite its limited outcome measures, these devices may be a cunning alternative to sort patients for a first-line reproductive care treatment strategy based on TMSC (Total Motile Sperm Count) or IMC (Inseminating Motile Count) after processing (Figure 5). With an IMC upon processing >1 million, it has been shown that it by- passes morphology as a single prognosis parameter, highlighting the influence of the IMC on pregnancy rates (Ombelet et al., 1997c; van Weert et al., 2004; Ombelet et al., 2014,2008b,^2003^; Thijssen et al., 2017; Punjabi et al., 2018; Saxena and Ghumman, 2019; Findeklee et al.,2020). Therefore, when reaching a threshold IMC of more than 1 million motile sperm after processing, IUI upon density gradient preparation and in combination with clomiphene citrate stimulation, can be proposed as a safe and cost-effective first-line infertility management strategy (Rutstein and Shah, 2004; Ombelet et al., 2008a,b; Ombelet et al., 2012, 2014; Esteves, 2014; Vargas-Tominaga et al., 2020). Alternatively to IUI and in the need of IVF, affordable simplified IVF may also be considered (Van Blerkom et al., 2014).

Prospectively, if endorsing the usability of these devices to resolve the current pitfalls of MSA assessment, guidelines for clinical implementation and follow-up of the performance of these devices should be coordinated by a capable and recognized entity such as the WHO. Finally, to be fully applicable as a point-of-care screening tool, more research needs to be done on the dependence on an internet connection and the usability of these devices with washed sperm. Importantly, further equipping these devices with morphology assessment and improving their accuracy by use of diagnostic intelligence (AI) and putative sperm dysfunction testing (i.e DNA fragmentation assessment, Hyaluronan Binding Assay and Seminal Oxydative Stress) capabilities, offering a complete and timely quantitative and qualitative overview of a sperm sample, are ideal add-ons to further develop and ensure clinical mainstay of these innovative devices (Agarwal et al., 2017; Dimitriadis et al., 2019, Hicks et al., 2019).

## Conclusion

Reliable and cost-effective sperm assessment is a requirement. Only with the widespread use of robust and accurate methods a clinical utility for MSA can be achieved. With evidence showing that implementation of continuous training and QC or the use of expensive CASA system, does not entirely exclude subjectivity and error, the use of commercially available, easy-to-use and cost-effective smart-phone-based simplified sperm assessment (volume, concentration and motility) for point-of-care male infertility screening is an actual alternative, provided that further clinical validation on neat and washed sperm is performed.

## Video scan (read QR)

Video 1: https://www.youtube.com/watch?v=cQMkPrnnqCc

**Figure:**
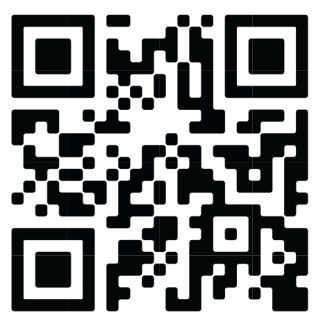


Video 2: https://www.youtube.com/watch?v=8gtvdAAYAnU

**Figure:**
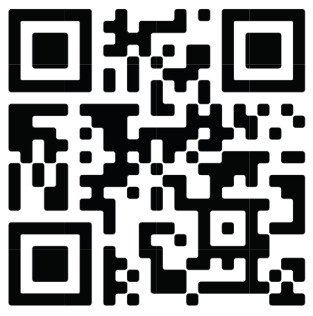


Video 3: https://www.youtube.com/watch?v=qCKGnfkqMA

**Figure:**